# The feasibility and acceptability of engaging older adults living with multiple long-term conditions, frailty, and a recent deterioration in health in research: Findings from the Lifestyle in Later Life – Older People’s Medicine (LiLL-OPM) study

**DOI:** 10.1186/s12877-024-05406-2

**Published:** 2024-10-14

**Authors:** Christopher Hurst, Lorelle Dismore, Antoneta Granic, Jane M. Noble, Susan J. Hillman, Miles D. Witham, Avan A. Sayer, Richard M. Dodds, Sian M. Robinson

**Affiliations:** 1 AGE Research Group, Translational and Clinical Research Institute, Faculty of Medical Sciences, Newcastle University, Newcastle upon Tyne, UK; 2 NIHR Newcastle Biomedical Research Centre, Newcastle upon Tyne Hospitals NHS Foundation Trust, Cumbria, Northumberland, Tyne and Wear NHS Foundation Trust and Faculty of Medical Sciences Newcastle University, Newcastle upon Tyne, UK; 3 Northumbria Healthcare NHS Foundation Trust, North Tyneside Hospital, Rake Lane, North Shields, Tyne & Wear, UK; 4 Department of Older People’s Medicine, Newcastle upon Tyne Hospitals NHS Foundation Trust, Newcastle upon Tyne, UK

**Keywords:** Multimorbidity, Frailty, Feasibility, Lifestyle, Diet, Physical activity

## Abstract

**Background:**

Older adults living with multiple long-term conditions (MLTC, also known as multimorbidity) and frailty are more likely to experience a deterioration in their health requiring specialist referral or hospital admission than individuals without these syndromes. However, this group of older people are underserved by research meaning that there is a limited evidence base for their care. This study therefore aimed (1) to determine if it is feasible to recruit and collect quantitative data to describe the health and lifestyle of older adults living with MLTC, frailty and a recent deterioration in health and (2) to assess if taking part in research is acceptable to this group of older adults.

**Methods:**

Participants were approached and recruited for this study via an Older People’s Medicine Day Unit in Newcastle upon Tyne, UK. The study took a mixed methods approach, involving quantitative and qualitative data collection. To determine the feasibility of carrying out research in this group, we quantified recruitment rate and collected data on the health and lifestyle, including diet and physical activity, of the participants. Qualitative semi-structured interviews were undertaken to assess acceptability. Two separate interviews were carried out focusing on involving older adults in research and the participants’ experiences of taking part in the research. Interviews were analysed using thematic analysis.

**Results:**

Fifty patients were approached to participate in the study with twenty-nine (58%) successfully recruited. It was feasible to collect information to describe the health and lifestyle of these older adults who demonstrated very low levels of physical activity. Participants reported that taking part in the research was acceptable to them with interview analysis generating three themes (1) developing a meaningful partnership, (2) enabling factors to participation: research at home with flexible delivery and (3) social and psychological benefits of research participation.

**Conclusions:**

It is feasible and acceptable to recruit and carry out research with this underserved group of older adults. Participants found taking part in this research to be acceptable and reported overall positive experiences of their involvement in the study and indicated that they would be willing to contribute to further research in the future.

## Introduction

Many older adults live with multiple long-term conditions (MLTC, also known as multimorbidity)—defined as the presence of two or more long-term health conditions, and frailty—a multi-system impairment associated with increased vulnerability to stressors [1, 2]. The coexistence of MLTC and frailty is common [3] and these health states interact to increase the risk of adverse health outcomes [4]. For example, older people living with the combination of MLTC and frailty have increased healthcare utilisation compared to individuals without these syndromes [5]. This group of older people therefore account for a substantial and growing proportion of encounters within clinical practice [6].

Individuals living with the combination of MLTC and frailty are more likely to experience a deterioration in their health requiring specialist referral or hospital admission than individuals without these syndromes [2]. Older people who have experienced a recent deterioration in their health are often excluded from clinical research, despite being at a point in their illness trajectory where they are most likely to interact with healthcare services and require evidence-based care [7, 8]. Including these older adults in research is important as taking part in clinical research can improve evidence-based care and lead to better outcomes for patients [9, 10].

Commonly cited barriers to participation in research are similar for older adults living with MLTC or frailty and include; health-related factors (e.g., pain, falls, fatigue, mobility problems), personal factors (e.g., lack of perceived benefit, daily routines) and research procedures (e.g., perceived burden of participation, travel requirements) [8–10]. However, less is known of the barriers to research participation in older adults living with the combination of MLTC and frailty who have experienced a recent deterioration in health—a group of patients whose unpredictable illness trajectory may make clinicians reluctant to include them in research [8]. Understanding the barriers which prevent this group engaging in research, as well as how to design and deliver research to meet the needs of this group, is necessary to develop the evidence base for their care [7].

Modifiable lifestyle factors, such as diet and physical activity, are associated with improved health outcomes in this population and represent important targets for intervention and behavioural change. For example, ’healthier’ dietary patterns have been associated with a lower frailty risk [11] with evidence that higher levels of habitual physical activity can delay the onset [12] and modify the progression of frailty [13]. Additionally, both dietary [14] and physical activity based interventions [15] can support recovery from acute illness and hospitalisation, as well as the associated deconditioning, that is commonly observed in this group of older adults [16]. Understanding if it is possible to assess diet and physical activity in this group of older adults will help to inform the development and implementation of future lifestyle interventions.

There remains a need to understand if older adults living with MLTC, frailty and a recent deterioration in health (identified in this study as an illness episode requiring interaction with a healthcare Day Unit Service) can be engaged in clinical research and how best to design and deliver research to meet the needs of this group. As such, the aims of this study were: (1) to determine if it is feasible to recruit and collect quantitative data to describe the health and lifestyle of older adults living with MLTC, frailty and a recent deterioration in health and (2) to assess if taking part in research is acceptable to this group of older adults.

## Method

### Study design

The Lifestyle in Later Life – Older People’s Medicine (LiLL-OPM) study was a mixed methods study including both qualitative and quantitative data collection [7]. The qualitative component of the LiLL-OPM study involved three semi-structured interviews focusing on (1) how to involve this group of older adults in research, (2) attitudes and barriers to resistance exercise training and (3) experiences of taking part in research. Data from the interview focusing on attitudes and barriers to resistance exercise are reported elsewhere [17]. Quantitative data collection included questionnaire-based health and lifestyle assessment and objective measurement of physical activity. The study was granted ethical approval from London – Harrow Research Ethics Committee (Ref 20/LO/1243) and received National Health Service (NHS) Health Research Authority approvals. All participants provided written informed consent and the study was conducted in accordance with the Declaration of Helsinki.

## Participants

Participants were recruited into the study from an Older People’s Medicine Day Unit service in Newcastle, UK. Patients are typically referred to this Day Unit for Comprehensive Geriatric Assessment (CGA), including medical, functional, mental health, social and environmental dimensions, because of a recent deterioration in their health (e.g., a fall, worsening mobility, unexplained weight loss). Patients were invited to participate in the study if they were living in their own home and had experienced a recent deterioration in health (defined as an illness episode requiring interaction with healthcare services) and referred to the Older People’s Medicine (OPM) Day Unit. Potential participants were provided with a Participant Information Sheet (PIS) and a brief explanation of the study by a clinician during their visit to the Day Unit. They were contacted by a member of the research team to discuss the study in greater detail after they had had time to consider their involvement. Participants were informed that the individual elements of qualitative and quantitative data collection were optional, and they could complete as many or as few as they wished. Informal carers (i.e., relative or friend, not a paid or professional carer) were invited to support participants during study assessments at the request of the participant. Any older adults who the OPM clinician felt were inappropriate to approach (e.g., those with moderate to severe dementia, or metastatic cancer with prognosis of only a few weeks) and those who were unable to provide informed consent, were excluded. There were no specific age criteria for inclusion in the study, although patients attending the OPM Day Unit are typically aged over 65 years. There was no upper age limit for inclusion in the study.

## Data collection

### Recruitment data

To evaluate if it was feasible to recruit participants living with MLTC, frailty and a recent deterioration in health to a research study, we recorded the total number of patients invited to take part in this study, the number of participants who were recruited into the study and drop-outs. For those who declined participation, we sought to ascertain the reasons.

## Health and lifestyle assessment

Participants were visited in their own home by an experienced researcher (CH, LD, RD) to complete a questionnaire-based health and lifestyle assessment. Data collected included questions on demographic information, living arrangements, medication usage, social support [18] and disability [19]. Frailty status was quantified using the Fried frailty score [11] and participants were asked to self-report the presence of long-term health conditions. The SARC-F (Strength, Assistance in walking, Rise from a chair, Climbing stairs, and Falls) questionnaire was used as a screening tool for sarcopenia [23].

Diet quality was assessed using a short food frequency questionnaire with participants asked to report the frequency of consumption of listed foods with responses used to calculate a diet quality score [20]. Higher diet quality scores indicate ‘heathier’ dietary patterns, characterised by higher consumption of fruit, vegetables, and wholegrain cereals. Participants were also asked to self-rate their overall diet (*“In general*,* how healthy is your overall diet?”*) [21]. Appetite was assessed using the Simplified Nutritional Appetite Questionnaire (SNAQ) [22] We characterised habitual physical activity in two ways: firstly, using the Rapid Assessment of Physical Activity (RAPA) questionnaire [23] and secondly using wrist-worn accelerometery. Participants were asked to wear a wrist-worn triaxial accelerometer 24 h/day for 7-days on their dominant wrist at a measurement frequency of 100 Hz (GENEActiv^®^ Original, ActivInsights Ltd, Kimbolton, UK). Data were processed and analysed using the R-package *GGIR* with average acceleration used as a proxy for total physical activity. Participants were provided with a paper diary to record any times of non-wear and the reasons why. Participants were asked to self-report smoking status and alcohol intake.

### Semi-structured interviews

The qualitative component of the LiLL-OPM study involved semi-structured interviews. We report data from semi-structured interviews with participants conducted at two time-points during the LiLL-OPM study: (1) to investigate how to approach and involve older adults in research and (2) to explore the participants experiences of taking part in the research. Interviews were conducted in participants’ homes or online (telephone/video call) using open-ended questions. The first interview focused on approaches to recruitment and data collection methods and aimed to understand more about how to approach and involve older adults in research. For example, *“We sometimes find it difficult to reach older adults living at home to invite them to take part in research. In your opinion how should we look to reach more older adults in the future to ask them to take part in research?”* and *“What would be the reasons that would prevent you from taking part in research and how can we help you overcome these?”*. The second, shorter, interview explored participants experiences of taking part in the study, for example *“‘Can you tell me what you liked/disliked about the study?”*. Interviews were audio-recorded and then deleted once transcribed verbatim. All participants were allocated a pseudonym.

### Data analysis

#### Health and lifestyle assessment

Health and lifestyle variables were characterised using descriptive statistics.

### Interviews

Data from the semi-structured interviews were analysed using reflexive thematic analysis (TA) whereby the researcher’s subjectivity is central to the analytical procedure. Meaning was therefore generated through interpretation of data, and saturation was subjective [24, 25]. Reflexive TA provides a rich and detailed, yet complex account of data. The data were analysed using an inductive approach with emergent themes grounded within the data. The six steps involved familiarisation with the dataset by reading and re-reading the data, to become immersed with its content. Identification of interesting aspects of the data relevant to the research question were documented using codes. This involved highlighting text (short segments of the data) throughout the data transcripts and coding as much data as possible to represent meaning and patterns within the data. Initial themes were generated by examining the codes and collating data to develop significant broader partners of meaning. The themes were constructed by the researchers (LD) subjectivity and an interpretative reflexive process. Themes were discussed and reviewed by two authors (LD and CH) by checking the themes against the coded data and entire dataset. Upon review of the themes, they agreed that the themes developed from the two data collection time points overlapped and are therefore presented within the manuscript collectively. Themes were defined as pattern of shared meaning underpinned by a central concept or idea. Themes were refined, defined, and named and finally, written up with supporting quotations.

## Results

### The feasibility of recruiting older adults living with MLTC, frailty and a recent deterioration in health

A total of 50 eligible patients who attended the OPM Day Unit were provided with a participant information sheet and an explanation of the study. After discussion with a member of the research team, 29 participants (58%) agreed to take part and were recruited into the study. The characteristics of these participants are described in Table 1. Reasons for declining study participation were: too unwell (*n* = 9), not interested or no specific reason (*n* = 6), unable to re-contact (*n* = 3), too busy (*n* = 1), concern over COVID-19 (*n* = 1) and hospitalised (*n* = 1).

**Table 1:** Participants’ characteristics

Characteristic	All (*n* = 28)	Men (*n* = 8)	Women (*n* = 20)
Age (years)	81 (7)	84 (6)	80 (7)
Ethnicity [n (%)]			
White British	26 (93)	8 (100)	18 (90)
Asian or Asian British – British Indian	1 (4)	0 (0)	1 (5)
Asian or Asian British – Pakistani	1 (4)	0 (0)	1 (5)
Accommodation status [n (%)]			
Standard housing (own home)	24 (86)	7 (88)	17 (85)
Sheltered housing with warden	3 (11)	1 (13)	2 (10)
Assisted living (extra care)	1 (4)	0 (0)	1 (5)
Social support [n (%)]^a^			
Lubben Social Network Scale ≥ 12 (not at risk)	16 (57)	3 (38)	13 (65)
Lubben Social Network Scale < 12 (at risk)	12 (43)	5 (63)	7 (35)

Values shown are Mean (SD) unless stated otherwise

^a^A score of less than 12 indicates an individual as being at risk for social isolation

**Fig. 1:**
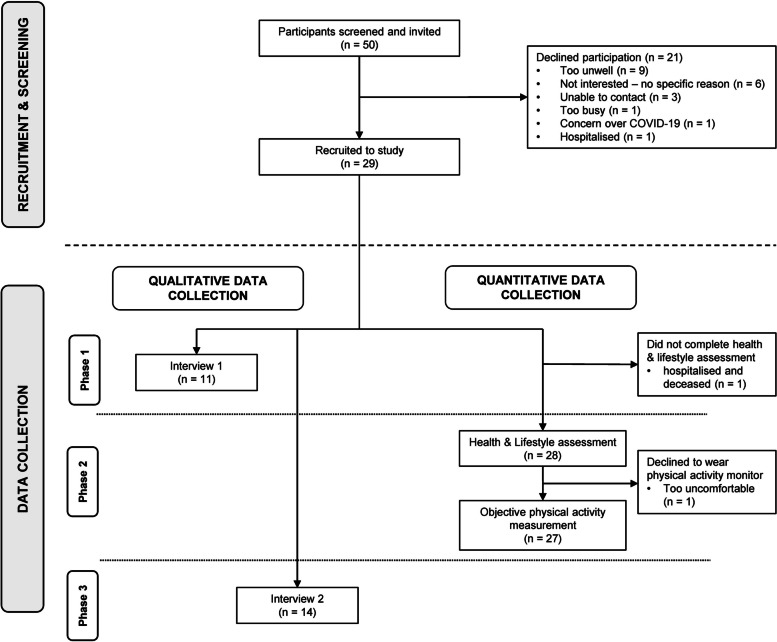
Data collection through the study

Of the 29 participants, 28 completed the health and lifestyle assessment and 14 took part in interviews; 10 participants completed every component of the quantitative and qualitative data collection (Fig. 1). No participants dropped out of the study. The quantitative health and lifestyle assessment took 50 (Standard deviation; SD 16) minutes to complete. Qualitative interview duration was 34 (SD 11) minutes and 11 (SD 4) minutes, for interview 1 and 2, respectively.

### The feasibility of collecting data to describe the health and lifestyle of older adults living with MLTC, frailty and a recent deterioration in health

Health and lifestyle data were collected from 28 participants who completed the quantitative health and lifestyle assessment. These data are presented in Tables 2 and 3.

**Table 2:** Health characteristics of participants

Characteristic	All (*n* = 28)	Men (*n* = 8)	Women (*n* = 20)
Number of long-term conditions [n (%)]			
0–1 (No MLTC)	2 (7)	2 (25)	0 (0)
≥ 2 (MLTC)	26 (93)	6 (75)	20 (100)
Number of medications [n (%)]			
0–4	6 (21)	3 (38)	3 (15)
≥ 5	22 (79)	5 (63)	17 (85)
Fried frailty score [n (%)]			
0 (Non-frail)	1 (4)	1 (13)	0 (0)
1–2 (Pre-frail)	7 (25)	1 (13)	6 (30)
3+ (Frail)	20 (71)	6 (75)	14 (70)
SARC-F [n (%)]			
0	1 (4)	1 (13)	0 (0)
1	2 (7)	0 (0)	2 (10)
2	1 (4)	0 (0)	1 (5)
3	1 (4)	0 (0)	1 (5)
4+	23 (82)	7 (88)	16 (80)
Disability			
Modified Barthel Index	89 (11)	92 (7)	88 (12)

Values shown are Mean (SD) unless stated otherwise

*SARC-F* Strength, Assistance, Rise, Climb – Falls questionnaire

**Table 3:** Lifestyle characteristics of participants

	All (*n* = 28)	Men (*n* = 8)	Women (*n* = 20)
**Diet**			
Diet quality score [Median (IQR)] ^a^	2.8 (1.2, 4.3)	1.3 (-2.4, 2.8)	3.4 (1.9, 5.1)
Self-rated diet quality [n (%)]			
Excellent	2 (7)	1 (13)	1 (5)
Very good	3 (11)	1 (13)	2 (10)
Good	13 (46)	3 (38)	10 (50)
Fair	7 (25)	3 (38)	4 (20)
Poor	3 (11)	0 (0.0)	3 (15)
SNAQ			
Not at risk (> 14)	14 (50)	5 (63)	9 (45)
At risk ( < = 14)	14 (50)	3 (37)	11 (55)
**Physical activity**			
RAPA aerobic activity score (1–7)	3.4 (1.7)	3.3 (1.9)	3.5 (1.6)
RAPA strength and flexibility score (0–3)	0.4 (0.9)	0 (0.0)	0.6 (1.1)
Total physical activity (m*g*)	16.5 (5.6)	11.4 (2.5)	18.7 (5.1)
**Smoking and Alcohol**			
Smoking status [n (%)]			
Never	11 (39)	2 (25)	9 (45)
Previous	14 (50)	6 (75)	8 (40)
Current	3 (11)	0 (0)	3 (15)
Alcohol consumption (units per week) [n (%)]			
0	20 (71)	4 (50)	16 (80)
1–10	5 (18)	2 (25)	3 (15)
> 10	3 (11)	2 (25)	1 (5)

Values shown are Mean (SD) unless stated otherwise

*IQR* Interquartile range, *SNAQ* Simplified Nutritional Appetite Questionnaire, *RAPA* Rapid Assessment of Physical Activity, *mg* milligravitational units

^a^ Higher values indicate higher quality diet

^b^ Higher values indicate higher levels of activity

#### Dietary assessment

All participants (*n* = 28) who undertook the health and lifestyle questionnaire were able to complete the food frequency questionnaire, enabling calculation of a diet quality score. The diet quality scores of women were higher when compared with men. However, diet scores did not correspond with the participants’ self-rated assessment (data not shown); 26% of men and 15% of women reporting their diets to be of excellent or very good quality (Table 2). Poor appetite was common, experienced by half (*n* = 14) the participants; the prevalence of poor appetite was higher among women.

#### Objective physical activity assessment

Of the 28 participants invited to wear a physical activity monitor, 27 (96%) agreed. Mean acceleration was 16.5 ± 5.6 m*g*. One participant declined to wear a monitor because of previous skin irritation at the wrist. All the participants wore the monitor every day across the 7-day period. Most participants made no comments on their accompanying paper diary and wore the physical activity monitor as instructed (i.e., 24 h per day for 7 days). One participant removed the physical activity monitor to shower as they found it more comfortable. One participant removed the watch for sleeping approx. 10pm – 8 am every day as they were feeling unwell.

#### Acceptability of taking part in research

Analysis of the interview data generated three themes: (1) developing a meaningful partnership, (2) enabling factors to participation: research at home with flexible delivery and (3) social and psychological benefits of research participation.

### Theme 1: Developing a meaningful partnership

Participants were positive about their experiences in the study and found that the recruitment and data collection procedures were acceptable. Factors such as altruism, curiosity and having the free time to take part were motivations for participating.

*“Well again if it helps people of my age in the future than I’m quite happy to have taken part”* (Male, Aged 74).

Participants emphasised that researchers should aim to develop meaningful partnerships with older adults as research participants. This involves treating older adults fairly, taking an interest in them and having a positive attitude toward them.

*“I know were all elderly…but I’ve been very impressed by the way in which*,* we haven’t simply been dismissed… I’ve been spoken to as somebody of equal standing… I think that’s very important the way in which people are treated…I think to be treated as a fair*,* opinion matters”* (Female A, Aged 81)

*“This is the first time in eighty-one years that I’ve ever heard anyone interested in the elderly… you know I’ve got to this age and nobody’s ever approached us. I’ve been elderly for quite a long time, so I mean I’m not expecting anything special.”* (Female B, Aged 81)

Taking time with older adults to explain the study procedures can help to alleviate any uncertainties and alter attitudes towards participation, which in turn will help aid recruitment and support the inclusion of older adults as research partners. Research teams should consider that a lack of understanding of what research involves may be a barrier to participation. Some individuals may be frightened to take part in a research study due to fear of the unknown.

*“I wouldn’t have done it if you hadn’t spoken to me on the phone*,* I wouldn’t have done it…” (Female*,* Aged 78)*

The relationship with the individual researchers was highly valued and the participants emphasised the importance of building rapport with the researcher. Having the opportunity to ask questions and having open discussions was important to support engagement in the study.

*“I must admit when you first came in the first time*,* you probably noticed I wasn’t as relaxed as I am today…I feel more at ease at answering…” (Female*,* Aged 90)*.

*“Things just come to your mind and you think ‘oh I can ask about that’ or just if you’ve got any queries or anything you can ask because you cannot always get talking to them at the hospital” (Female*,* Aged 77)*.

The interpersonal skills of members of the research team and the strategies involved in communicating with potential participants, are of great importance to support engagement in a research study.

### Theme 2: Enabling factors to participation: Research at home with flexible delivery

Conducting research visits at home was identified as being important both for recruiting and retaining participants. Home visits are convenient and comfortable and can reduce the barriers associated with attending hospital for research activities. These barriers included travel costs, use of public transport and reliance on informal carers for support.

*“I would prefer my own home…well I would have to arrange a taxi…I mean I could do that if that was easier for you but I’m just thinking of me*,* it’s handy” (Female*,* Aged 84)*.

*“I wouldn’t have been able to take part if you hadn’t been able to come to my home*,* so that was a terrific plus*,* so that was one of the reasons that I was able to do it really (Female*,* aged 79)*.

Despite this however, some older adults suggested that they would be willing to attend hospital for research purposes.

*“Just if there was any travelling…I don’t mind doing it…but if I can avoid travelling in anyway…I cannot get around…I cannot walk very far*,* short distances…I’ve got a scooter*,* I’ve got a walking stick…but I can’t go very far…I get a taxi…it costs me a fortune*,* I pay for taxi’s” (Male*,* Aged 87)*.

Research visits need to be organised around older adults’ routines and there needs to be flexibility in research appointments. Some participants expressed a preference for afternoon visits because of needing time in the morning to take medication and feeling more physically able later in the day. Some older adults have a heavy reliance on informal carers and the availability of carers will impact on potential participation.

*“Because it gives us time*,* first thing in the morning I’ve got to take my time and of course my husband as well*,* but I go so slow in the morning until my engine starts to work a bit…so that’s why I think if we can get it into the afternoon then I’m better” (Female*,* Aged 79)*.

*“I’d have to take the wheelchair; I mean I can walk about the house but if I go out*,* I’ve got to go in the wheelchair…I cannot do very much*,* no because I’ve got to hold onto something the whole time…and my eyesight as well*,* not good” (Female*,* Aged 90)*.

Participants described the assessments within the present study as being convenient, but there was variability in what was perceived to be acceptable in terms of the length of research visits (i.e., twenty minutes to a couple of hours).

*“It’s possible for a short visit twenty minutes yes*,* it’s possible once a week*,* as long as it doesn’t clash with dental*,* chiropodist*,* other doctors’ appointments*,* hospital appointments” (Female*,* Aged 92)*.

Potential barriers to research participation included health-related issues such as, mobility problems (e.g., experiencing dizziness, problems with balance), a fear of falling and communication difficulties (e.g., issues with hearing or eyesight).

*“It’s just my balance*,* so standing without an aid maybe*,* I wouldn’t feel very comfortable with that I don’t think*,* you see I don’t use my stick or anything in the house very much*,* but I seem to sort of lose it…I think it’s the confidence that’s gone*,* that’s what’s gone” (Female*,* Aged 79)*.

*“Well*,* I do have a fear…when I’m walking*,* I do have falls sometimes. So*,* that’s why you know I’m very*,* very careful…” (Female*,* Aged 69)*.

Health related problems resulted in a lack of confidence to attend appointments independently, with informal carers playing a key role in supporting their relatives.

#### Theme 3: Social and psychological benefits of participation

Participants felt that participating in the research promoted social and psychological benefits. They enjoyed spending time with the researchers and looked forward to the research visits. Participants described a sense of receiving social support through their participation and they felt that by engaging in the research it improved their mood.

*“It’s nice to open the door to see a nice friendly face standing there and you’ve just opened my eyes a little bit to the world again with me being so stuck indoors and not seeing anybody…its nice having the company” (Female*,* Aged 78)*.

*“Because it was company as well…just talking really*,* one to one… you as a person…in fact I look forward to seeing you…” (Female*,* Aged 81)*.

The participants felt valued and gained a sense of purpose from taking part in the research. They felt positive about being involved and able to contribute to the study.

“*well*,* you get a bit of self*,* self what would you call it satisfaction that you’ve done something you’ve spared the time*,* other people’s come to see you and it’s all for the benefit of the whole community it’s not just yourself* “ (Male, Aged 86).

*“It’s nice to see that you can still contribute…sometimes it is the small things in life that count… you’re doing something positive*,* something positive coming out of it… this has made me feel quite positive again it’s sort of picked me up a little bit” (Female*,* Aged 78)*.

Importantly, participants indicated that they would be willing to be involved in further research in the future.

*“I would*,* if I was well enough I would do it yeah without doubt”* (Male, Aged 86).

“… *this has made me feel quite positive again its sort of picked me up a little bit*,* yes*,* I would*” (Female, Aged 78).

Overall, the participants experienced positive outcomes through their taking part in the research.

## Discussion

Older adults living with MLTC, frailty and a recent deterioration in health are underserved by research, resulting in a limited evidence base for their care [26]. Designing research that meets the needs of this group of older adults is necessary to fill this evidence gap. Our mixed-methods study has shown that (1) it is feasible to recruit and collect quantitative data to describe the health and lifestyle of older adults living with MLTC, frailty and a recent deterioration in health; (2) taking part in research is acceptable to this group of older adults. These important findings will support the design and delivery of future research involving this population.

A range of personal and structural barriers to research participation exist for older adults living with MLTC or frailty [8]. These barriers are likely to be exacerbated in those living with the combination of MLTC and frailty who can have an increasingly uncertain and less stable health trajectory. However, we have shown that it is feasible to recruit older adults living with MLTC, frailty and a recent deterioration in health, and this group of older adults are willing and able to participate in clinical research. Encouragingly, 58% of the participants who were invited to take part in the study were recruited. It may be that our approach to introducing a research opportunity during a usual clinical interaction was valuable. Previous work has suggested that healthcare providers with an already established link to the patient may enhance recruitment to clinical studies [27] with older people perceiving invitations from those involved in their care to be more meaningful [28]. These data provide useful context when planning recruitment for future studies involving this population group.

Lifestyle factors including physical targeted physical activity [29, 30] and dietary interventions [11, 31] have potential to improve markers of health and function in older people and can support recovery from acute illness and associated deconditioning [32]. As such, there is a need to be able to assess these behaviours to evaluate relationships with health outcomes and identify opportunities for intervention. In this study, we were able to successfully describe physical activity and diet in a group of older adults living with MLTC, frailty and a recent deterioration in health – demonstrating the feasibility of data collection and use of tools that can be employed in future studies involving this population group. While questionnaire-based assessment of physical activity is inexpensive and relatively easy to administer, objective device-based assessment provides a more reliable measure by eliminating recall bias [33]. We found that wrist-worn accelerometery was feasible and acceptable to our participants. Our participants engaged in low levels of physical activity although there was meaningful between participant variation. Our data indicate that this group of older people are more active than hospitalised older adults [34] but much less active than community dwelling older adults [35]. We were able to collect data using different assessment tools on self-rated appetite and diet quality and to administer a short food frequency questionnaire. While data interpretation is limited in this small study, it is encouraging that we established the feasibility of these data collection methods in this population group. Our data also suggest that there is meaningful variability in diet within this group of older adults. The low levels of physical activity and the variation in diet observed, illustrate the need for further investigation of these behaviours in this population group. The methods we have used to do this in the current study can be employed in future work with older adults living with MLTC, frailty and a recent deterioration in health.

Findings from our qualitative interviews demonstrated that participants found taking part in this research to be acceptable. Participants reported positive experiences of the study, including the recruitment and data collection methods used, and indicated that they would be willing to contribute to future research. Altruism was a common motivator for enrolling in this study, a finding reflected more widely for research participation in older people [36]. A key factor in the successful engagement of participants in this project was the development of a meaningful partnership between the participant and the researcher based on trust and mutual respect. This finding mirrors previous work showing that genuine reciprocal relationships between patients and researchers are key to engaging older adults successfully in research [37]. Participants felt valued by being listened to and appreciated the opportunity to engage with the researcher during the study visits. This highlights the importance of increased personal contact by researchers sensitive to the unique needs of older adults to support research participation [38].

Previous work has suggested that taking part in research can provide older adults with a break from everyday life and can be a way of alleviating frail older adults feelings of loneliness [39]. However, there is considerable variation in how frail older people want, and can be involved in research [39] and research teams should seek to provide various opportunities to reflect this. This could include patient and public involvement and engagement activities as well as enrolment into patient registries which can offer future research opportunities [40]. Many older people want to participate in research [27], a desire reflected by our participants, who indicated that one of the reasons they agreed to take part in the study was simply because they were asked. This highlights a clear need to provide accessible opportunities for older adults living with MLTC, frailty and a recent deterioration in health to contribute to research.

The success of the present study provides important learning for future research involving this group of older adults. We found that difficulties travelling to research appointments, and the associated costs, as well as health related problems (e.g., fear of falling, poor mobility, communication difficulties) and a reliance on support from informal carers would be substantial barriers to research participation. Strategies to overcome these barriers should be embedded into the design of future research. For example, home visits are convenient and comfortable and should be offered wherever practically possible. In this study we made a considerable effort to minimise the burden of research participation, by ensuring our approach was flexible and adaptive to individual needs (e.g., research visits completed at home organised around participants’ daily routines). The effectiveness of this approach is evidenced by the high proportion of participants that completed every component of the study data collection and the fact that no participants dropped out during the study. Minimising participant burden and ensuring a flexible and adaptive approach appear to be fundamental to the successful delivery of research in this population group [8, 37].

### Strengths and limitations

A major strength of this study is that the participants were recruited from an Older People’s Medicine Day Unit where research opportunities are usually limited, particularly for those with a recent deterioration in health. We have successfully managed to recruit and carry out research with a group who are underserved by research and are reflective of those who are seen in clinical practice. Our findings provide important learning that could support future work in this population group. Another strength of this work was that the qualitative interviews were conducted by a health psychologist (LD) with considerable experience in carrying out semi-structured interviews with older adults. However, it is acknowledged that our study sample, which were recruited from a single Day Unit, were predominantly female and of white British ethnicity. In addition, we did not include older people who were unable to provide informed consent in the study, meaning that we may have missed out on important perspectives from these individuals. There remains more to be done researching this topic, and future research should attempt to understand the perspectives of consultees for adults with incapacity, as well as exploring the views of patients on the use of consultees, to support future and ongoing participation in research [41].

## Conclusion

We have shown that it is feasible to recruit and carry out research with older adults living with MLTC, frailty and a recent deterioration in health. We were able to successfully collect data health and lifestyle data from these participants and importantly our approaches to recruitment and data collection, including wearable devices, were acceptable. A personal and flexible approach should be incorporated into the design of future research involving older adults living with frailty, MLTC and a recent deterioration in health who remain underserved by research and represent an important group for inclusion in future research studies.

## Data Availability

The datasets generated and analysed during the current study are not publicly available due to participant privacy and confidentiality. De-identified data is available from the corresponding author on reasonable request.

## Abbreviations

CGA: Comprehensive Geriatric Assessment
LiLL-OPM: Lifestyle in Later Life-Older People’s Medicine
MLTC: Multiple long-term conditions
NHS: National Health Service
OPM: Older People’s Medicine
PIS: Participant Information Sheet
RAPA: Rapid Assessment of Physical Activity
SARC-F: Strength, Assistance, Rise, Climb – Falls
SNAQ: Simplified Nutritional Appetite Questionnaire
TA: Thematic analysis
